# Beneficial Effects of Musicality on the Development of Productive Phonology Skills in Second Language Acquisition

**DOI:** 10.3389/fnins.2020.00618

**Published:** 2020-07-07

**Authors:** Franco Delogu, Yi Zheng

**Affiliations:** ^1^Department of Humanities, Social Sciences and Communication, Lawrence Technological University, Southfield, MI, United States; ^2^Department of Psychology, Stony Brook University, Stony Brook, NY, United States

**Keywords:** Arabic, auditory working memory, musicality in language, AMMA, productive phonology, second language processing

## Abstract

Previous studies show beneficial effects of musicality on the acquisition of a second language (L2). While most research focused on perceptual aspects, only few studies investigated the effects of musicality on productive phonology. The present study tested if musicality can predict productive phonological skills in L2 acquisition. Sixty-three students with no previous exposure to Arabic were asked to repeatedly listen to and immediately reproduce short sentences in standard Arabic. Before the sentence reproduction task, they completed an auditory discrimination task in three different between-subjects condition: attentive, in which participants were asked to discriminate phonological variations in the same Arabic sentence that they were asked to reproduce later; non-attentive, in which participants were asked to detect beeps in the same Arabic sentences without paying attention to their phonological content; and no-exposure, in which participants performed the discrimination task in another language (Serbian). The first, third and seventh reproductions of each participant were rated for intelligibility, accent, and syllabic errors by two independent evaluators, both native speakers of Arabic. Primary results showed that the intelligibility of the reproduced sentences was higher in participants with high musicality scores in the Advanced Measures of Music Audiation. Moreover, the intelligibility of sentences produced by highly musical participants improved more over time than the intelligibility of participants with lower musicality scores. Previous exposure to the Arabic sentence was beneficial in both the attentive and non-attentive conditions. Our results support the idea that musicality can have effects on productive skills even in the very first stages of L2 acquisition.

## Introduction

This study focuses on the analysis of the relationships between musicality and speech production skills in L2 acquisition in adults. More specifically, we focus on the association between musicality and the acquisition of very early phonological and prosodic abilities in a second language.

L2 proficiency requires perceptual and productive competencies. Perceptual-receptive competencies include the understanding of lexical and grammatical structures and the detection of phonological contrasts and prosodic cues in the new language. Productive-motor competencies include the ability to compose and produce correct words and sentences in the L2, to produce the correct phonemes, and to produce the correct prosody. Until recently, receptive abilities attracted the most interest of psycholinguistic and cognitive neuroscience research, whereas productive abilities were less investigated. The limited attention of research on the productive aspects of L2 learning proficiency is probably due to the fact that the receptive and productive abilities are believed to be strongly associated. However, several studies found no correlation between the participants' ability to produce and to perceive a given contrast, a segment, or a consonant sequence (Golestani and Pallier, 2007; Kabak and Idsardi, 2007; Park, 2011; Kalathottukaren et al., 2017). Moreover, the evidence of possible different degrees of competence in receptive (passive) and productive (active) bilingualism is consistent with the existence of a gap between the understanding and the production of a second language (e.g., Umbel et al., 1992; Wei et al., 1992).

A limitation of most of the previous studies on L2 productive phonology is the rather heterogeneous competencies of participants, which varies for native language background and the amount of exposure and experience with the target L2. Because the amount of L2 exposure is very hard to control, even in those studies that select the sample from a homogenous population with the same number of years of immersion in the linguistic context, in this study we tested participants on a language in which they had no previous experience and provided a controlled exposure of the target L2 language. With such an approach, we sought to measure the association of factors such as musicality, working memory capacity, and passive and active exposure to the target L2 with the early acquisition of productive phonological and prosodic skills in an unknown L2.

What is the role of musical expertise and musicality in the acquisition of productive phonological skills in an L2? The understanding of the nature of the associations between these inherently human cognitive domains helps shed light on the evolution of the mechanisms of human communication in general. According to Patel's OPERA theory, musical training facilitates language processing due to the overlaps of brain regions that process language and music, and to the great demand of *precision, emotion, repetition*, and *attention* during music training (Patel, 2010, 2012). Studies have shown that musicians outperformed non-musicians in extracting prosodic information from speech in a familiar language, as well as in a foreign language (Thompson et al., 2003, 2004). However, in contrast with perceptual studies, in which the association between musical skills and second language acquisition has been amply demonstrated (e.g., Delogu et al., 2006, 2010; Magne et al., 2006; Slevc and Miyake, 2006; Marie et al., 2011), the evidence of a positive association between musical skills and L2 productive skills is still debated.

A quantifiable index of productive proficiency is foreign accent (FA), which is often defined as the pronunciation of a language that shows deviations from native standards. Recurrently identified factors influencing L2 phonological skills in general and FA in particular (see Piske et al., 2001 for a review) are the length of residence in an L2-speaking country, gender, formal instruction, motivation, language learning aptitude, amount of native language use, and L1 background. The association between musicality and FA is still a matter of debate. On the one side, there are studies in which musicality failed to correlate with FA (Tahta et al., 1981; Flege et al., 1995). Similarly, Posedel et al. (2012) showed that the number of years of musical training does not predict the pronunciation quality in L2 Spanish. On the other side, Slevc and Miyake (2006) showed a beneficial effect of musical ability on phonological proficiency in L2 English. Pastuszek-Lipinska (2008) showed that musicians outperform non-musicians in shadowing foreign-language sequences in six different languages. Similarly, Stegemööller et al. (2008) suggested that music training facilitates vocal production of speech and song. Also, Milovanov et al. (2010) have shown that university students with higher musical aptitude have a better L2 pronunciation than students with less musical aptitude. More recently, results consistent with an association between musicality and L2 phonological proficiency were obtained using the accent faking paradigm (Coumel et al., 2019).

These contrasting results are probably associated to the variability of methods used across studies to operationalize both the influential factor (musicality) and the dependent variable (productive skills). In some cases, musicality is operationalized as years of musical training (e.g., in Posedel et al., 2012), whereas in other cases as the result of a musicality test (e.g., in Slevc and Miyake, 2006). Likewise, productive skills are also differently measured, whether as verbal shadowing of foreign languages (e.g., Pastuszek-Lipinska, 2008), as amount of FA (e.g., Flege et al., 1995), as the ability to imitate FAs (Christiner and Reiterer, 2013; Reiterer et al., 2013; Coumel et al., 2019), or as the reproduction accuracy of L2 words and sentences (Slevc and Miyake, 2006). Another factor that can cause variability of results is the amount of expertise in (or exposure to) the targeted L2. The amount of previous experience in the language, in fact, is often considered as one of the most important influential factors in phonological proficiency (Flege and Liu, 2001). In this study, as anticipated earlier, we control the influence of previous exposure to the target language by using participants with no previous experience with the target language, and we measure the effect of a controlled exposure on a productive phonology task.

This study aimed at testing several factors able to predict the acquisition of productive skills in second language acquisition. We focused in particular on the influence of musicality. We also took into account the influence of gender, short-term memory capacity, and presence and quality of previous exposure to the target L2. Operatively, we measured the ability of our participants to reproduce a set of sentences in a foreign language after exposing them (or not) to the target language in a discrimination task that preceded the productive task and that required them to pay attention (or not) to the phonological features of the target language.

## Methods

### Overview

The study included two linguistic tasks: a discrimination task in which participants had to decide if two consecutive sentences in a foreign language were identical or not and a productive task in which participants were asked to listen and reproduce sentences in a foreign language. The experiment also included the Advanced Measures of Music Audiation (AMMA; Gordon, 1989), which is a musicality test that assesses melodic and rhythmic skills, and a digit span memory test, which provided a measure of short-term memory capacity. We decided to use a measure of musicality such as AMMA, instead of a measure of music training (e.g., number of years of formal musical training) for different reasons. First, while music training does not necessarily reflect the actual and current state of musical abilities of participants, a musicality measure such as AMMA provides a quantitative score of musicality on the very day that the L2 task is performed. Second, AMMA allows the inclusion of participants regardless of their musical training and daily hours of practice, allowing a wider generalizability of the results.

### Participants

A total of 63 students from Lawrence Technological University (mean age = 23.60 years, SD = 11.13 years; 32 were female) participated in the study. All participants were native speakers of English with no self-reported auditory impairment conditions and no previous competence or extended exposure to Arabic or Serbian languages. None of them were bilingual. They were randomly assigned to three groups, each of which received different conditions in a same–different linguistic discrimination task. They all received either monetary compensation or course credits for their participation.

### Materials and Tasks

A total of 96 sentences, 48 in Arabic and 48 in Serbian, were recorded by native speakers of Arabic and Serbian. For each language, the recorded sentences were evenly divided between male and female speakers. The variant of Arabic used in this study is modern standard Arabic, which is used in many countries in educational contexts and in the media. Sentences were specifically constructed for this experiment with the following constraints: they were all between 9 and 11 syllables long and have an average duration of ~2 s. Specifically, the Arabic sentences had an average duration of 2.18 s (SD = 0.29 s), and the Serbian sentences had an average duration of 2.13 s (SD = 0.33 s). The average duration of the sentences in the two languages was not significant [*F*_(1, 46)_ = 0.17, *p* = 0.68]. Most of the sentences in both languages had the same syntactic subject–verb–object or subject–verb–adverb structure. An example of an Arabic sentence is the following: “Alsafar Moreh Bealtaya Rah” (al-sa-far mo-reh be-al-ta-ya rah) [English translation: Traveling by plane is comfortable]. As the meaning of the sentence was not relevant for our experimental goals, the sentences in Arabic and Serbian were not translated from the same English sentences. The complete list of the sentences can be found in Appendix 1 in Supplementary Material. After recording, alterations were applied to single syllables of each of the 96 sentences, so that for each sentence there existed an original version, a pitch-alteration version, a time-alteration version, and a pure tone–added version, for a total of 384 stimuli. Pitch- and time-altered versions were included to operationalize, in a same–different recognition task, two main sources of prosodic variations (pitch and time) that are also crucial in music. The pure-tone version was added to create a condition in which the detection task does not require the participant to put specific attention in the phonological and prosodic dimensions of the sentences. More details about the recognition task will be provided in the description of the procedure. An example of an original sentence, pitch-altered version, time-altered version, and pure tone–added version are available as additional media materials. In order to avoid participants focusing only on specific parts of a sentence in the same–different recognition task, the syllable in which the alteration was applied varies in different sentences. A total of 80 sentences (including original, pitch-, time-, and pure tone–altered sentences) were used as experimental materials, whereas 16 sentences were used in the training sessions.

In the pitch condition, the pitch contour of a single syllable (F0) was altered by transposing the syllable up two or three semitones in order to create weak (two semitones) and strong (three semitones) alterations, respectively (see Schön et al., 2004, for a similar paradigm). The amount of pitch alteration was tested in a pilot study with 10 non-musicians in which the strong alteration was correctly detected 85% of the time and the weak alteration 72% of the time. In the time condition, the duration of a single syllable was altered by stretching the syllable by either 75 or 100% in order to create weak and strong time alterations, respectively. The detectability of time alterations was tested in a pilot study with 10 non-musicians in which the strong alteration was correctly detected 80% of the time and the weak alteration 74% of the time. A third alteration condition was built by inserting a 100-ms-long pure tone in a syllable of each sentence. The pitch of the tone was always 6% (approximately one semitone) higher than the F0 of the syllable's vowel to which the tone was overlapped. Two conditions of detectability were created: an easy condition, in which the loudness of the tone was 10% softer than the perceived loudness of the vowel, and a difficult condition, in which the loudness of the tone was 20% softer than the perceived loudness of the vowel.

### Procedure

#### Same-Different Recognition Task

Participants listened to two consecutive sentences separated by 1 s of silence. Their task was to indicate if the two sentences were identical or different by pressing either a “same” key or a “different” key on a keyboard. The same keys were used in all three between-subjects conditions to indicate that the two sentences were identical (“same”) or to indicate that a variation occurred (“different”). The same–different recognition task was intended to provide participants with a controlled amount of exposure to the target language (Arabic) before they perform the productive task. The same–different discrimination was performed in three conditions, defined as *attentive* (provides an attentive exposure to Arabic), as *non-attentive* (provides a non-attentive exposure to Arabic), and as *control* (does not provide any exposure to Arabic). The conditions varied according to a between-subject design, where each group of subjects performed only one of the conditions of the experiment. In the *attentive* condition, the second sentence could differ from the first in the pitch or duration of a single syllable. In the non-attentive condition, the second sentence could differ from the first because of a brief beep contained in one of the syllables. We must clarify that by labeling the condition as “non-attentive,” we specifically refer to the lack of attention toward the prosodic and phonological information. Still, the auditory stimuli in general must be attentively attended in order to detect the presence of brief beeps during the same–different discrimination task. In the control condition, the task is identical to the one in the attentive condition, but it is performed in a different language (Serbian) than the one (Arabic) to be reproduced in the productive task. The goal of the three conditions in the discrimination tasks was to manipulate the presence and the type of exposure to the language to be reproduced. First, in the *attentive* condition, participants were requested to attentively listen to the sentences in order to detect syllabic variations. Furthermore, because the same sentences they later reproduced in the productive task were used, the participants had the occasion to practice with the sounds and the sentences of the to-be-reproduced language while performing the discrimination task. In fact, while listening, they had to focus on the phonological and prosodic aspects of the sentences in order to detect possible variations between the first and the second sentence. By contrast, in the non-attentive beep condition, participant listened to the same sentences as in the attentive condition, but they did not have to focus on phonological and prosodic content in order to determine variations. In fact, as they simply have to detect the presence of a beep in the second sentence, their task was limited to the detection of a target signal (a pure tone) embedded in an unknown language. In the third, control condition, the attentive task was performed in a different language (Serbian) than the one they have to reproduce, with the consequence that there was no exposure to the language they will reproduce in the productive task.

#### Productive Task

For each sentence, participants listened to the recording twice and then reproduced it twice. In order to reduce as much as possible the F0 distance between models and reproductions, male participants listened and reproduced the male version of the native-speaker recordings, whereas female participants listened and reproduced the female version of the native-speaker recordings. In the first reproduction, participants repeated the sentence along with the native-speaker recording, whereas in the second reproduction they produced the sentence aloud without the recording. The second reproduction was recorded via a microphone. For each sentence, this process was sequentially repeated seven times. After seven recorded reproductions of a sentence, the participant started listening to and reproducing a new sentence until each of 10 different sentences was reproduced seven times for a total of 70 recordings per each participant. The order of presentation of the 10 sentences was randomized across participants.

#### Digit Span

A computerized version of the digit span forward task was used to determine participants' digit span. Sequences of single digit spoken numbers from one to nine were presented, and participants were required to repeat the sequence aloud. The sequence length began with four numbers, and every two sequences increased in length by one. The sequence presentation ended when the participant made two consecutives mistakes. The longest sequence repeated without making two consecutive mistakes determined the participants' span.

#### Advanced Measures of Music Audiation

AMMA (Gordon, 1989) measures the ability to detect tonal and rhythmic variations in a pair of musical fragments. The test includes 30 experimental trials plus three practice trials. For each trial, participants chose between tonal variation, rhythmic variation, and no variation.

### Analysis

The testing phase produced 4,410 audio recordings of sentences spoken by participants (63 participants ^*^ 10 sentences ^*^ 7 reproductions). Two independent evaluators were recruited to perform a perceptual analysis of the distance between the spoken reproductions and the models. As there is evidence that judges perform better when they are bilingual themselves (Scovel, 1977; Beardsmore, 1980; Flege and Hillenbrand, 1984), we selected two native speakers of Arabic that were also fluent speakers of English. The evaluators were two senior students at Lawrence Technological University: one, a 20-year-old female student of psychology born and raised in Jeddah, Saudi Arabia, and the other, a 21-year-old student of Mechanical Engineering born and raised in Damascus, Syria. Both evaluators used standard modern Arabic daily in school contexts. The evaluators assessed three different aspects of sentence reproduction proficiency: (a) intelligibility, that is, how much of the sentence was comprehensible. This dimension is measured as the percentage of the message that is understood by the evaluator. (b) Accuracy, that is, number of errors. This dimension is measured by the ratio of number of incorrectly reproduced syllables over the total number of reproduced syllables. (c) Foreign accent: evaluators were asked to rate the strength of FA from 0 to 10, where 0 indicates indistinguishable from a native speaker, and 10 indicates an extremely strong FA.

Productions 1, 4, and 7 of each sentence from each participant were evaluated by the two evaluators for intelligibility, correctness, and accent. The interrater agreement was calculated with intraclass correlation coefficient (ICC). Intraclass correlation coefficient values are reported in Table 1.

**Table 1:** Intraclass correlation coefficients (ICCs) between the ratings of the two evaluators.

	**Intelligibility**	**Phonological errors**	**Foreign accent**
ICC 1st reproductions	0.82	0.56	0.65
ICC 4th reproductions	0.88	0.53	0.63
ICC 7th reproductions	0.87	0.52	0.74

According to widely accepted guidelines (Cicchetti, 1994), the ICC between our two raters expressed an excellent interrater agreement for intelligibility, a good agreement for accent, and a fair agreement for correctness. We ran an analysis of variance (ANOVA) with gender, previous exposure, and AMMA as between-subjects variables and learning as a within-subject variable. As described above, previous exposure has three levels [attentive, non-attentive, control (Serbian)].

## Results

In a preliminary analysis, the digit span scores showed no correlation with any of the three dependent variables at any of the learning stages of the productive task (Table 2).

**Table 2:** Correlation coefficient (Pearson *R*) and relative *p*-values between the digit span scores and each measure of the productive task.

	**Intelligibility**	***p***	**Syllabic errors**	***p***	**Accent**	***p***
First reproduction	−0.07	0.57	0.16	0.21	−0.04	0.74
Fourth reproduction	−0.04	0.65	0.07	0.61	−0.05	0.78
Seventh reproduction	−0.05	0.68	0.03	0.83	−0.07	0.62

Considering the absence of correlation with any of the dependent variables of the main design and in order to keep low the number of factors, we decided to exclude digit span from the list of factors of the main analysis.

Participants were classified in two different groups according to their score in the AMMA test. Participants who scored equal to or lower than the median value of the distribution of AMMA scores (median = 14) were included in the low AMMA group, whereas participants scoring higher than the median value (≥15) were included in the high AMMA group.

The main results are summarized in Table 3.

**Table 3:** Summary of averages, standard errors and group comparison of intelligibility, phonological errors, and accent scores in the two AMMA groups.

		**1st Reproduction**	**4th Reproduction**	**7th Reproduction**
Intellegibility	Low AMMA	0.77 (0.15)	1.27 (0.20)	1.6 (0.23)
	High AMMA	1.32 (0.14)	2.35 (0.18)	2.91 (0.21)
	Significance	*p = 0.036*	*p < 0.0001*	*p < 0.0001*
Accent	Low AMMA	5.05 (0.18)	5.63 (0.17)	5.98 (0.18)
	High AMMA	5.33 (0.17)	6.03 (0.16)	6.44 (0.16)
	Significance	*p > 0.05*	*p = 0.025*	*p = 0.013*
Phonological errors	Low AMMA	4.80 (0.26)	4.66 (0.28)	4.55 (0.32)
	High AMMA	5.57 (0.23)	4.58 (0.25)	4.23 (0.29)
	Significance	*p > 0.05*	*p > 0.05*	*p > 0.05*

*Italics indicate significant p values*.

Concerning intelligibility, results showed that the AMMA was significant, *F*_(1, 51)_ = 13.41, *p* = 0.0006, η^2^ = 0.21, indicating that the high AMMA group (mean = 2.24, SE = 0.19) outperformed the low AMMA group (mean = 1.20, SE = 0.20) in the intelligibility scores. Previous exposure was also significant, *F*_(2, 51)_ = 5.12, *p* = 0.009, η^2^ = 0.16. *Post-hoc* analysis [Fisher least significant difference (LSD)] showed that the active Arabic discrimination group (mean = 2.22, SE = 0.24) had a greater intelligibility score than the Serbian discrimination group (mean = 1.19, SE = 0.28, *p* = 0.003), but did not differ from the passive Arabic group (mean = 1.84, SE = 0.22, *p* = 0.26). The passive Arabic group had significantly higher intelligibility scores than the Serbian group (*p* = 0.049). Gender was also significant, *F*_(1, 51)_ = 5.67, *p* = 0.02, η^2^ = 0.10, indicating that female participants (mean = 2.05, SE = 0.20) scored higher in intelligibility that the male ones (mean = 1.39, SE = 0.19). Learning was significant, *F*_(2, 102)_ = 131.92, *p* < 0.0001, η^2^ = 0.72. *Post-hoc* analysis (Fisher LSD) showed that the seventh reproduction of the sentence (mean = 2.31, SE = 0.17) was more intelligible than the fourth reproduction (mean = 1.86, SE = 0.15, *p* < 0.001), as well as the first reproduction (mean = 1.08, SE = 0.12, *p* < 0.001). The first reproduction was scored as less intelligible than the fourth reproduction (*p* < 0.001).

**Figure 1:**
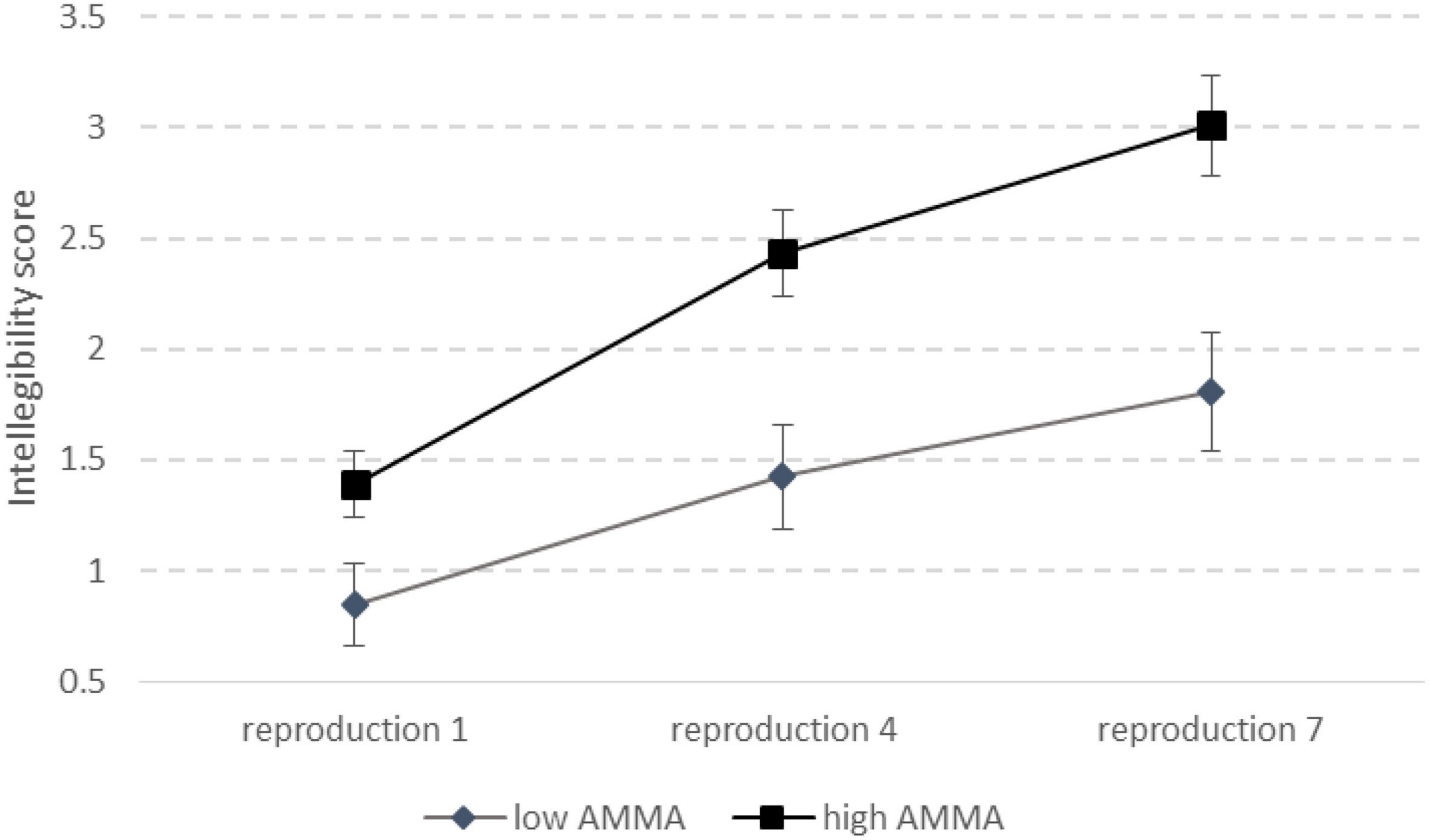
Rates of intelligibility as a function of musicality and reproduction attempts.

The interaction between learning and AMMA (Figure 1) was significant, *F*_(2, 102)_ = 12.733, *p* < 0.0001, η^2^ = 0.199. *Post-hoc* analysis (Fisher LSD) showed that the *p*-values progressively decreased when comparing low and high AMMA in the first (*p* = 0.034), the fourth (*p* = 0.00015), and the seventh reproduction (*p* < 0.00001), indicating that the difference in intelligibility in the low and high AMMA groups progressively increased with the repetitions.

The interaction between learning and gender was significant, *F*_(2, 102)_ = 4.73, *p* = 0.011. *Post-hoc* analysis indicated that while the intelligibility scores of the first reproduction of female participants were not different from the ones of the male participants (*p* = 0.09), the difference became significant in reproduction 4 (*p* = 0.005) and reproduction 7 (*p* = 0.0005). The interaction between learning and previous exposure was not significant, *F*_(4, 102)_ = 1.64, *p* = 0.169. Also, the interaction between gender and AMMA was not significant, *F*_(1, 51)_ = 0.237, *p* = 0.628, as well as the interaction between gender and previous exposure, *F*_(2, 51)_ = 0.03, *p* = 0.97. The interaction between previous exposure and AMMA was not significant, *F*_(2, 51)_ = 0.391, *p* = 0.68. All of the three-way interactions and four-way interactions were not significant.

Concerning accent*, learning* was significant, *F*_(2, 102)_ = 146.2, *p* < 0.00001, η^2^ = 0.74. *Post-hoc* analysis (Fisher LSD) showed that FA in the seventh reproduction (mean = 6.27, SE = 0.12) was significantly weaker (*p* < 0.001) than in the fourth (mean = 5.88, SE = 0.11) and in the first production (mean = 5.24, SE = 0.11, *p* < 0.001). Additionally, the first reproduction had more accent than the fourth reproduction (*p* < 0.001). Gender was not significant, *F*_(1, 51)_ = 2.14, *p* = 0.14. AMMA was not significant, *F*_(1, 51)_ = 2.87, *p* = 0.096, indicating that participants with low musicality scores (mean = 5.46, SE = 0.17) and participants with high musicality scores (mean = 5.79, SE = 0.14) did not significantly differ in their accent performance. Previous exposure was also not significant, *F*_(2, 51)_ = 2.81, *p* = 0.069, indicating that the active Arabic group (mean = 5.79, SE = 0.19), passive Arabic group (mean = 5.69, SE = 0.17), and Serbian group (mean = 5.39, SE = 0.21) did not vary significantly in their accent scores. The interaction between previous exposure and gender was significant, *F*_(1, 51)_ = 3.99, *p* = 0.024. *Post-hoc* analysis showed that female participants outperformed male participants in accent only in the condition where they were not exposed to Arabic, but to Serbian (*p* = 0.0018), whereas there were no gender differences in the quality of accent in the active (*p* = 0.31) or passive (*p* = 0.66) conditions. All of the other interactions were not significant.

Concerning accuracy (syllabic errors), learning was significant, *F*_(2, 96)_ = 32.081, *p* < 0.0001, η^2^ = 0.400. *Post-hoc* analysis (Fisher LSD) showed that in the seventh reproduction of the sentence (mean = 4.23, SE = 0.22) the number of phonological errors committed was significantly less (*p* = 0.0098) than in the fourth (mean = 4.59, SE = 0.20) as well as in the first (*p* < 0.0001). Additionally, in the first reproduction (mean = 5.49, SE = 0.17) participants committed more errors than the fourth reproduction (*p* < 0.0001). AMMA was not significant, *F*_(1, 48)_ = 0.274, *p* = 0.603, η^2^ = 0.0057, with the low musicality group (mean = 4.86, SE = 0.27) scoring with equivalent levels of accuracy as the high musicality group (mean = 4.68, SE = 0.22). Perceptual type was not significant as well, *F*_(2, 48)_ = 0.699, *p* = 0.502, η^2^ = 0.028, as the active Arabic group (mean = 4.54, SE = 0.30), the passive Arabic group (mean = 4.69, SE = 0.26), and the Serbian group (mean = 5.07, SE = 0.34) did not significantly differ in number of errors. Regarding gender, female participants (mean = 4.62, SE = 0.24) and male participants (mean = 4.92, SE = 0.26) did not differ significantly in their amount of errors, *F*_(1, 48)_ = 0.757, *p* = 0.389, η^2^ = 0.016.

The interaction between AMMA and learning was significant, *F*_(2, 96)_ = 4.01, *p* = 0.02, η^2^ = 0.08. *Post-hoc* analysis (LSD Fisher) showed that among members of the high AMMA group, there was a progressive decrease in errors in successive attempts at reproducing the sentence. *p* < 0.001, 0.0287, and < 0.001 indicate that the differences between the first and fourth, fourth and seventh, and first and seventh reproductions, respectively, were all significant. In contrast, in the low AMMA group, the difference between the fourth and seventh reproductions was not significant (*p* = 0.147). A significant difference appeared only between the first and fourth reproductions and between the first and seventh reproductions, with the following *p*-values, respectively: *p* = 0.007 and *p* = 0.00006. All of the other interactions were not significant.

As we assessed the association between musicality and L2 phonological production by splitting the sample in two groups according to their performance in the AMMA test, we could potentially incur in reliability issues. In fact, as pointed out by MacCallum et al. (2002), an artificial dichotomization of a continuous variable such as AMMA could open the field to a number of issues, including loss of power and effect size and the risk of overlooking non-linear effects. In order to reduce such risks, we calculated the correlation between the continuous scores of AMMA and the intelligibility, phonological errors, and accent scores in their three longitudinal measures (first, fourth, and seventh reproduction). As shown in Table 4, the results of the correlation analysis provide analogous results as the ANOVA. It is worth noting that the correlation coefficients for intelligibility and accent increase progressively during the reproduction task.

**Table 4:** Correlation between AMMA scores and production measures in the 1st, 4th, and 7th reproductions.

	**Intel. 1**	**Intel. 4**	**Intel. 7**	**Errors 1**	**Errors 4**	**Errors 7**	**Accent 1**	**Accent 4**	**Accent 7**
AMMA	0.3139	0.4307	0.4670	0.2289	0.0442	−0.0308	0.3378	0.4189	0.4646
*p*	*p* = 0.012	*p* = 0.000	*p* = 0.000	*p* = 0.071	*p* = 0.731	*p* = 0.811	*p* = 0.007	*p* = 0.001	*p* = 0.000

## Discussion

In this study, we investigated the influence of several factors on L2 speech phonological and prosodic competencies. Specifically, we tested whether musicality, gender, and previous exposure to the linguistic materials influence the intelligibility, accuracy, and level of FA of speech reproductions of Arabic sentences.

Concerning musicality, results showed that musicality is associated with a greater accuracy in sentence reproduction in an unknown language. In fact, the sentences produced by highly musical participants were significantly more intelligible than the sentences produced by participants with lower musicality scores. It is also notable that highly musical participants tended to improve more with a short amount of training. This result provides new evidence of music–language domain interactions. As far as we know, no previous study tested whether musicality can be associated with the accuracy of the reproduction of spoken sentences in an unknown language. If we consider the association between music and language in general, this result is consistent with previous evidence of music-to-language transfer effects and in particular on L2 acquisition (Slevc and Miyake, 2006; Delogu et al., 2010; Marie et al., 2011).

The role of musicality on the amount of FA is not completely clear in our results. In fact, while the main factor AMMA is not significant, there is a trend for less-accented reproduction by highly musical group in the fourth and the seventh reproductions. Such trend is confirmed by the progressively stronger correlation between the continuous measure of AMMA and the accent scores in the first, fourth, and seventh reproductions (Table 4). Such contrasting findings, possibly influenced by the numerosity of the sample, are in line with conflicting evidence in the literature. Specifically, whereas some previous studies did not find an association between musicality or musical expertise and levels of FA (Tahta et al., 1981; Flege et al., 1995; Posedel et al., 2012), other studies found evidence of the influence of musicality on prosodic aspects in second language acquisition (Slevc and Miyake, 2006) and of an association between musical aptitude and better foreign language pronunciation skills in adults (Milovanov et al., 2010). The lack of an effect is perhaps linked to a combination of methodological factors such as the reduced sample size and the not-so-high interrater agreement for the accent measure.

Additionally, the level of musicality of the participants did not influence correctness, measured as the number of syllabic errors. The lack of evidence of an influence is probably due to the difficulty of reporting the number of syllabic errors from the two evaluators, who were not formally trained in linguistics. Such difficulty is expressed by their interrater agreement for syllabic errors estimation, which is the lowest of the three measures.

Regarding prior exposure to the sentences, participants who performed an active or passive perceptual test on the same Arabic sentences produced more intelligible sentences than participants who performed an active perceptual test in another language (Serbian). Interestingly, the groups who performed the active Arabic task (phonological discrimination) and the groups who performed a passive Arabic task (beep detection) did not differ in the level of intelligibility of their sentence reproduction. These results could indicate that previous exposure to the language is a beneficial factor for the production of more intelligible sentences not only when a pre-reproduction exposure is focused on the phonological and prosodic features of the sentence to be reproduced, but also when the listener's attention is diverted toward the detection of near-threshold intrusive signals embedded within the sentence. An alternative interpretation that we currently cannot exclude is that the processing of the Serbian sentences in the same–different recognition task could have created an interference effect that made it more difficult for participants to later reproduce sentence in Arabic. Previous exposure to the sentences did not have an influence on the amount of accent or on the amount of syllabic errors committed.

Gender was important for accent. Consistent with previous evidence (Asher and García, 1969; Tahta et al., 1981; Thompson, 1991), women showed less FA than men. Interestingly, women with low musicality displayed a lower level of FA when compared with men in the same low-musicality group. However, in the high musicality group, the two genders did not differ. This result suggests that the advantage of women over men in accent/prosody/speech (Hyde and Linn, 1988; Buckner et al., 1995; Soleman et al., 2013) can be compensated by musicality. Female and male participants' reproductions did not differ in intelligibility or number of syllabic errors committed.

Performance at the digit span task did not show any significant association with any of the three language production measures. As many studies demonstrate the influence of working memory in language acquisition (Ellis, 1996; Mackey and Sachs, 2012; Williams, 2015), this finding was unexpected. A possible reason is that the digit span forward test we used does not provide an estimate of working memory processing, but only of its capacity. Our productive task could be particularly demanding in working memory processing and not very challenging in terms of capacity. However, it is worth reporting that many findings indicate that short-term memory measures do predict SLA. For example, Ellis (1996) illustrates experiments that use digit span (as used in a previous version of the ITPA) as a successful predictor SLA. Additionally, the PSTM used by Mackey and Sachs (2012) involves a similar procedure to the digit span as a measure of phonological short-term memory.

Intelligibility, accent and syllabic accuracy of the reproduced sentences markedly improved during the course of the productive task. It is of particular importance to note that the amount of improvement observed in both intelligibility and syllabic accuracy was significantly modulated by the musicality of the subjects. In particular, in their first attempt at speech reproduction, participants, regardless of their AMMA score, performed similarly in terms of intelligibility. Crucially, in the fourth and seventh reproductions, although all participants improved, those with high musicality showed greater improvement than those with low musicality.

In conclusion, the results of this study support the hypothesis that musicality can be a beneficial factor in the acquisition of productive skills in a second language even in the very first stages of L2 acquisition, when the imitation of the phonological and prosodic contents of the unfamiliar language is a challenging task for any learner. Not only do people with higher musicality produce more intelligible sentences, but also the comprehensibility of their sentences improves significantly more over time when compared with the sentences produced by people with lower musicality. It should be clarified that our study does not test L2 acquisition *per se*, but simply investigates the ability to reproduce foreign language sounds, which is only a limited aspect of L2 acquisition. Also, the evidence of an association between musicality and sentence reproduction accuracy in an unknown language does not in itself provide evidence of a transfer effect from musicality to language acquisition, as there is still the possibility of underlying unknown factors causing musicality scores and the quality of sentence reproductions to covary. Nevertheless, such evidence of an association between musicality and the accuracy of sentence reproduction supports the idea of the beneficial effects of integrating musical education in second language acquisition pedagogy.

## Data Availability Statement

The datasets generated for this study are available on request to the corresponding author.

## Ethics Statement

The studies involving human participants were reviewed and approved by IRB of Lawrence Technological University. The patients/participants provided their written informed consent to participate in this study.

## Author Contributions

FD wrote the manuscript and conducted the experimental study. FD and YZ performed the statistical analysis and reviewed the paper. All authors contributed to the article and approved the submitted version.

## Conflict of Interest

The authors declare that the research was conducted in the absence of any commercial or financial relationships that could be construed as a potential conflict of interest.
