# Supplementary material for: Beneficial Effects of Musicality on the Development of Productive Phonology Skills in Second Language Acquisition
Source: Front Neurosci. 2020 Jul 7;14:618. doi: 10.3389/fnins.2020.00618 (PMC7358579; doi:10.3389/fnins.2020.00618)
Supplement: Supplementary file 1 [file Table_1.docx]

**24 Sentences in Arabic**

| 1 | al | sa | far | Mo | reh | Be | al | ta | ya | Rah |  |
| --- | --- | --- | --- | --- | --- | --- | --- | --- | --- | --- | --- |
|  | رة | يا | ط | ال | با | ريح | م | فر | س | ال |  |
| 2 | La | zem | ko | la | Na | ne | kon | met | aw | ne | in |
|  | ين | ون | عا | مت | ون | نك | نا | كل | زم | لا |  |
| 3 | Al | ha | yah | hel | wa | be | al | ma | ha | bah |  |
|  | بة | مح | ال | با | وة | حل | ياة | ح | ال |  |  |
| 4 | Mn | Al | Da | ro | ry | ek | mal | al | de | ra | sah |
|  | سة | را | د | ال | مال | اك | ري | رو | الض | من |  |
| 5 | Al | sa | da | qah | maa | na | ha | al | se | dq |  |
|  | دق | ص | ال | ناها | مع | قة | دا | ص | ال |  |  |
| 6 | Al | ea | lah | ho | ma | sha | ms | al | ha | yah |  |
|  |  | ياة | ح | ال | مس | ش | ما | ه | ئلة | عا | ال |
| 7 | Al | se | ha | min | ah | am | ko | Noz | al | don | ya |
|  | يا | دن | ال | نوز | ك | م | اه | من | حة | ص | ال |
| 8 | Al | wa | tan | en | ti | maa | le | kol | sha | khs |  |
|  |  | خص | ش | لك | ل | ماء | ت | ان | نط | و | ال |
| 9 | Al | sa | lam | min | asa | se | yat | al | am | an |  |
|  |  | ن | اأم | ال | يات | س | أسا | من | لا | س | ال |
| 10 | Al | om | ak | bar | md | ra | sa | lel | ha | yah |  |
|  |  | ياة | ح | لل | سة | ر | مد | بر | أك | أم | ال |
| 11 | Min | Al | gha | baa | mo | ra | fa | qat | al | say | ean |
|  | ئين | سي | ال | ق | ف | را | م | باء | غ | ال | من |
| 12 | la | ti | tho | m | kol | sha | ey | ma | yea | je | bak |
|  |  | بك | ج | يع | ما | يء | ش | كل | م | تذ | لا |
| 13 | mar | ha | lat | al | tha | na | we | ya | ja | mee | lah |
|  | لة | مي | ج | ية | و | ن | ث | ال | لة | ح | مر |
| 14 | La | tez | lem | tra | daa | wat | al | maz | lum | say | eah |
|  | ئة | سي | لوم | مظ | ال | وة | دع | ترا | لم | تظ | لا |
| 15 | La | tem | shi | maa | as | hab | al | soo | aba | dan |  |
|  |  | داً | اب | سوء | ال | حاب | أص | مع | شي | تم | لا |
| 16 | Al | elm | nor | wa | al | ja | hl | za | lam |  |  |
|  |  |  | لام | ظ | هل | ج | ال | و | نور | علم | ال |
| 17 | al | aab | ak | bar | ha | nan | le | kol | bi | nt |  |
|  |  | نت | ب | كل | ل | نان | ح | بر | أك | أب | ال |
| 18 | al | okh | wan | ez | wa | wo | fa | khr | wo | hob |  |
|  |  | حب | و | خر | ف | و | وة | عز | وان | أخ | ال |
| 19 | al | sha | ha | da | sa | nad | le | kol | sha | khs |  |
|  |  | خص | ش | كل | ل | ند | س | دة | ها | ش | ال |
| 20 | al | ha | yat | saa | bah | la | ken | ja | mee | lah |  |
|  |  | لة | مي | ج | كن | ل | بة | صع | ياة | ح | ال |
| 21 | al | ta | fa | oal | eh | sas | ra | ea | je | dan |  |
|  |  | داً | ج | ئع | را | ساس | اح | ؤل | فا | ت | ال |
| 22 | kha | lek | sa | ead | al | ha | yat | hel | wah |  |  |
|  |  |  | وة | حل | ياة | ح | ال | عيد | س | ليك | خ |
| 23 | Ne | som | min | al | sa | bah | ha | ta | al | mgh | reb |
|  | رب | مغ | ال | تى | ح | باح | ص | ال | من | مصو | ن |
| 24 | mi | ta | ha | tet | kha | Raj | min | al | jam | aah |  |
|  |  |  | عة | مجا | ال | نم | رج | خ | حتت | تى | م |

**24 Sentences in Serbian**

1. Ma-ma je do-šla da vi-di be-bu.
2. Muž mi je ku-pi-o cve-će da-nas.
3. Po-gle-da-ceš od-lič-nu pred-sta-vu!
4. Ri-ba je pli-va-la u je-ze-ru.
5. Su-tra pro-da-jem ku-ću na la-kat!
6. No-sim džem-per kad je vr-lo hlad-no.
7. Nju-ška pra-se-ta je stal-no mo-kra.
8. Ta ko-lev-ka se lju-lja na ve-tru.
9. Na-sta-vnik je po-če-o ka-o đak.
10. Fen-jer se u-ga-si-o sa ju-trom.
11. Ča-mac plo-vi re-kom ka o-ba-li.
12. Naš konj i-ma-o je cr-nu gri-vu.
13. Te že-ne i-ma-ju le-pu ko-su.
14. Li-va-da je ze-le-na u je-sen.
15. Zveč-ka je ba-če-na na žu-ti pod.
16. Tvo-je ve-li-ke o-či su tuž-ne.
17. Ja pi-jem ra-ki-ju sva-ko ju-tro.
18. Nje-ne ci-pe-le su ka-o čam-ci.
19. Hleb te či-ni si-tim za do-ru-čak.
20. Zi-ma je bi-la sa pu-no sne-ga.
21. Ći-lim će kra-si-ti na-su sobu.
22. Do-sta mi je pi-sa-nja pi-sa-ma.
23. Po-sle po-sla u gra-du je gu-žva.
24. Re-če-ni-ce srp-skog ni-su te-ške.
